# Differential outcomes of Zika virus infection in *Aedes aegypti* orally challenged with infectious blood meals and infectious protein meals

**DOI:** 10.1371/journal.pone.0182386

**Published:** 2017-08-10

**Authors:** Yan-Jang S. Huang, Amy C. Lyons, Wei-Wen Hsu, So Lee Park, Stephen Higgs, Dana L. Vanlandingham

**Affiliations:** 1 Department of Diagnostic Medicine/Pathobiology, College of Veterinary Medicine, Kansas State University, Manhattan KS, United States of America; 2 Biosecurity Research Institute, Kansas State University, Manhattan KS, United States of America; 3 Department of Statistics, College of Arts and Sciences, Kansas State University, Manhattan, KS, United States of America; CEA, FRANCE

## Abstract

**Background:**

Infection of mosquitoes is an essential step for the transmission of mosquito-borne arboviruses in nature. Engorgement of infectious blood meals from viremic infected vertebrate hosts allows the entry of viruses and initiates infection of midgut epithelial cells. Historically, the infection process of arboviruses in mosquitoes has been studied through the engorgement of mosquitoes from viremic laboratory animals or from artificial feeders containing blood mixed with viruses harvested from cell cultures. The latter approach using so-called artificial blood meals is more frequently used since it is readily optimized to maximize viral titer, negates the use of animals and can be used with viruses for which there are no small animal models. Use of artificial blood meals has enabled numerous studies on mosquito infections with a wide variety of viruses; however, as described here, with suitable modification it can also be used to study the interplay between infection, specific blood components, and physiological consequences associated with blood engorgement. For hematophagous female mosquitoes, blood is the primary nutritional source supporting all physiological process including egg development, and also influences neurological processes and behaviors such as host-seeking. Interactions between these blood-driven vector biological processes and arbovirus infection that is mediated via blood engorgement have not yet been specifically studied. This is in part because presentation of virus in whole blood inevitably induces enzymatic digestion processes, hormone driven oogenesis, and other biological changes. In this study, the infection process of Zika virus (ZIKV) in *Aedes aegypti* was characterized by oral exposure via viral suspension meals within minimally bovine serum albumin complemented medium or within whole blood. The use of bovine serum albumin in infectious meals provides an opportunity to evaluate the role of serum albumin during the process of flavivirus infection in mosquitoes.

**Methods:**

Infectious whole blood meals and infectious bovine serum albumin meals containing ZIKV were orally presented to two different groups of *Ae*. *aegypti* through membrane feeding. At 7 and 14 days post infection, infectious viruses were detected and viral dissemination from gut to other mosquito tissues was analyzed in orally challenged mosquitoes with 50% tissue culture infectious dose method on Vero76 cells.

**Results/Conclusions:**

Zika virus infection was significantly impaired among mosquitoes orally challenged with infectious protein meals as compared to infectious whole blood meals. These results indicate the importance of the blood meal in the infection process of arboviruses in mosquitoes. It provides the basis for future studies to identify critical components in the blood of vertebrate hosts that facilitate arbovirus infection in mosquitoes.

## Introduction

Hematophagy emerged multiple times during the evolution process of arthropod species [1]. Acquisition of blood meals from vertebrate hosts provides the nutrients and energy to ensure reproductive success of hematophagous arthropods. In addition to the importance in the reproduction of arthropods, the behavior of hematophagy plays an integral role in the transmission of pathogens as the infection in arthropod vectors is often initiated through the engorgement of infectious blood meals from vertebrate hosts that have developed systemic infections. Mosquitoes are responsible for millions of arbovirus transmissions to humans and animals every year. Although other modes of transmission, such as transovarial transmission and non-viremic transmission, contribute to the maintenance of arboviruses, infection of arboviruses in mosquitoes initiated by the feeding of mosquitoes on viremic blood undoubtedly has the highest public health significance because of the involvement of infected vertebrate hosts [2–5].

To study the infection process of arboviruses in mosquitoes, the most widely used approach is to allow the engorgement of infectious blood meals from various sources. Different techniques have been developed to induce the engorgement of blood meals, including the use of viremic animals, membrane feeders, and cotton pledgets [6–9]. This method of feeding has generated an incredible amount of knowledge on vector-virus interactions [10,11]. In addition to the infection established through the engorgement of infectious viruses, numerous physiological responses can also be induced by the ingestion of blood meals from the vertebrate hosts. These responses not only facilitate the digestion of blood but also enable the detoxification of metabolic byproducts in mosquitoes and the production of neuroendocrines required for vitellogenesis and the completion of gonotrophic cycles [12–14]. These additional responses to the blood meal indicate the need to further characterize this process in order to better understand how the infection of arboviruses and digestion of blood meals can interact with each other.

In contrast to the limited understanding of the interactions between blood digestion and arbovirus infection, there have been several formulations of protein meals developed to study the digestion of blood meals in the past two decades. Formulas of artificial diets were initially developed to replace blood meals for egg production and often are based on different mixtures of proteins and other molecules dissolved in buffered salt solution without the presence of cellular components [15,16]. Recently, it has been demonstrated that cell-free protein meals containing one single type of protein, bovine serum albumin (BSA), alone is sufficient to support the completion of gonotrophic cycle in *Aedes aegypti* and *Ae*. *albopictus* [17,18]. Ingestion of protein meals can trigger the secretion of proteases, which resembles the digestive process of blood meals. Interestingly, meals containing purified cellular materials such as erythrocytes and purified erythrocyte ghosts failed to support egg production and do not stimulate the production of digestive proteases [17,19]. Therefore, it has become increasingly clear that different components present in the blood of vertebrate hosts can induce different digestive responses in mosquitoes. Using protein meals with defined chemical formulations as a method to deliver infectious viruses, the relationship between the digestion of blood meals and the infection of arboviruses can be further determined.

In this study, a protein meal based on BSA solution was used to study the infection of Zika virus (ZIKV) in *Ae*. *aegypti*. BSA was chosen as the protein component of the infectious meal for mosquitoes because of the high concentration of serum albumin in the blood of various mammalian hosts and the conserved sequences and structures among serum albumin among various species [20]. The formulation was chosen based on previous success in supporting the completion of the gonotrophic cycle in both *Ae*. *aegypti* and *Ae*. *albopictus* [17,18]. Our results demonstrated the infection process of ZIKV is significantly impaired when infectious viruses are delivered through the ingestion of infectious protein meals. These results highlight the importance of cellular materials present in the blood meal in facilitating the infection process in mosquitoes and show the feasibility of using protein meals to investigate vector-virus interactions in the future.

## Materials and methods

### Cells and viruses

Vero76 cells were used in this study for the propagation of virus stocks and detection of infectious viruses in homogenized tissues and whole mosquitoes as previously described [21]. The PRVABC59 strain of ZIKV was originally isolated from an infected individual in Puerto Rico in 2015 and passaged three times prior to this study. The stock used in this study was prepared by inoculating monolayers of Vero76 cells followed by the harvest of virus stocks when 70–80% cytopathic effect was observed. Cellular debris was removed by centrifugation at 1,200 r.p.m. for 5 minutes at 4˚C. Virus stocks were stored at -80 ˚C until used for *per os* challenge.

### Mosquitoes and per os infection

The Higgs white-eye strain of *Ae*. *aegypti* (F>20) was used for *per os* infection in this study. Details of the study have been described in our previous publications [22,23]. Seven-to-ten-day-old mosquitoes were deprived of sugar for 24 hours prior to challenge with ZIKV using the Hemotek feeding system (Discovery Workshop). Two types of infectious meals, infectious whole blood meals (WBMs) and infectious bovine serum albumin meals (BSAMs), were prepared for *per os* challenge in different groups of mosquitoes. Infectious WBMs were generated by mixing equal volumes of defibrinated sheep blood (Colorado Serum Company) and virus stocks. Infectious BSAMs were prepared by the equal volume mixture of Dulbecco’s phosphate buffered saline supplemented with BSA at 250 mg/ml and virus stocks. Mosquitoes designated as negative control groups received blood meals and protein meals containing culture media mixed with defibrinated sheep blood and BSA in Dulbecco’s phosphate buffered saline solution, respectively. All blood meals and protein meals were supplemented with ATP at 3 mM as a phagostimulant. The final concentration of BSA in protein meals at 125 mg/ml was chosen in this study based on previous publications showing its capability of supporting egg production in *Ae*. *aegypti* and *Ae*. *albopictus* [17,18].

Mosquitoes were allowed to ingest the infectious WBMs and BSAMs for one hour. Titers of infectious WBMs and BSAMs containing ZIKV used for the oral challenge are summarized in Table 1. Engorged individuals were sorted based on the size of abdomen and returned to cartons after feeding. Three mosquitoes from each carton were sampled and frozen immediately after feeding to estimate the quantity of infectious viruses engorged through feeding. At 7 and 14 days post infection (d.p.i.), mosquitoes were collected by mechanical aspiration and cold anesthetized. A subset of mosquitoes was dissected to separate the abdomen section and the secondary tissues such as the head, thorax, wings, and legs of individual mosquitoes to identify the disseminated form of infection. The other subset of mosquitoes were collected without dissection to assess the replication kinetics of ZIKV initiated through the engorgement of infectious blood meals and protein meals. The numbers of mosquitoes sampled at 7 and 14 d.p.i. are listed in Table 1.

**Table 1:** Average titers of ZIKV infectious meals and engorged mosquitoes and the numbers of mosquitoes sampled at 7 and 14 d.p.i.

Group	Average titers of infectious meals (logTCID_50_/ml)	Average titers of engorged mosquitoes	0 d.p.i.	7 d.p.i.		14 d.p.i.	
			Engorge whole mosquitoes sampled	Dissected mosquitoes	Whole mosquitoes	Dissected mosquitoes	Whole mosquitoes
WBMs	6.7±0.5	4.6±0.8	9	23	16	30	14
BSAMs	7.0±0.2	4.4±0.8	9	28	19	22	13

To determine the status of infection, homogenates of dissected tissues or whole mosquitoes were titrated using the 50% tissue culture infectious dose (TCID_50_) method. Infection rates were determined by calculating the numbers of positive detected in any of the titrated samples divided by the total numbers of mosquitoes tested in each group at each time point. The disseminated form of infection was defined as the positive detection of infectious viruses in any secondary tissues. The incidence was determined using the numbers of positive samples of secondary tissues divided by the numbers of infected mosquitoes dissected in each group.

### Statistical analysis

Infection rates and dissemination rates between mosquitoes receiving infectious blood meals and infectious protein meals at comparable titers were analyzed by Fisher’s exact test. Comparison of replication kinetics among two different groups of mosquitoes was performed by Student’s *t*- test. All statistical analyses were performed with SAS Software.

## Results

### Efficiency in the establishment of infection by infectious blood meals and infectious protein meals

To evaluate the importance of different components in the blood of vertebrate hosts in the infection process of arboviruses, infectious WBMs and BSAMs containing ZIKV were used to determine if the ingestion of infectious blood meals and infectious cell-free BSAMs can lead to the difference in the efficiency in the establishment of infection. Two groups of *Ae*. *aegypti* were challenged with infectious WBMs and BSAMs at comparable titers. Similar titers indicated mosquitoes ingested a comparable amount of infectious viruses regardless of the meals used to deliver the infectious viruses.

ZIKV infection was found to be established by *per os* infection with infectious WBMs and BSAMs as summarized in Table 2. Infection of ZIKV in *Ae*. *aegypti* established by the engorgement of infectious WBMs led to the infection rates at 59.0% (23/39) and 68.2% (30/43) at 7 and14 d.p.i. (*p* = 0.36), respectively. Similarly, there was no distinguishable difference in the infection rates of ZIKV among mosquitoes exposed to infectious BSAMs at 7 (23.4%, 11/47) and 14 (31.4%, 11/35) d.p.i. (*p* = 0.46).

**Table 2:** Infection rate of ZIKV in *Ae*. *aegypti* receiving infectious blood meals and protein meals at 7 and 14 d.p.i.

Group	7 d.p.i.	14 d.p.i.
WBMs	59.0% (23/39)	68.2% (30/43)
BSAMs	23.4% (11/47)	31.4% (11/35)

At both 7 and 14 d.p.i., infection rates of ZIKV were consistently higher among mosquitoes receiving infectious WBMs compared with those receiving infectious BSAs as shown in Table 2. At 7 d.p.i., infection rate of ZIKV in the group of mosquitoes presented with infectious WBMs was significantly higher when the virus was presented in infectious BSAMs (59.0% vs. 23.4%, *p* = 0.001). At 14 d.p.i., the infection rate of mosquitoes receiving infectious WBMs remained consistently higher at (68.2%) than the infection rate observed among those receiving infectious BSAMs (31.4%) (*p* = 0.0013).

Our observations indicate that the establishment of ZIKV infection in *Ae*. *aegypti* through the engorgement of infectious WBMs was significantly more efficient than through the engorgement of infectious BSAMs. Because BSA composes greater than 50% of blood plasma proteins in healthy human adults, our results can provide the basis for understanding how soluble protein materials can play a role in the establishment of arbovirus infection in mosquitoes and indicates that blood cells and other soluble molecules present in the blood of vertebrate hosts may play a more critical role in the infection of ZIKV in *Ae*. *aegypti*.

### Delivery of viruses by infectious protein meals does not affect viral dissemination or replication in mosquitoes

Since the delivery of viruses by infectious blood meals and protein meals showed a significant difference in efficiency for the establishment of ZIKV in *Ae*. *aegypti*, the next logical question to address is whether or not the low infectivity of ZIKV caused by the ingestion of infectious BSAMs can affect viral replication and dissemination into other tissues from the infected midgut. To determine the incidence of the disseminated form of infection, tissues from the thorax, head, wings, and legs of individual mosquitoes were examined to detect infectious virus. Replication kinetics of ZIKV at 7 and 14 d.p.i. was determined by titrating infected whole mosquitoes.

Dissemination rates of ZIKV among *Ae*. *aegypti* orally challenged with infectious WBMs and BSAMs at 7 and 14 d.p.i. are summarized in Table 3. Dissemination rates of ZIKV significantly increased from 50.0% (7/14) at 7 d.p.i. to 100.0% (21/21) in *Ae*. *aegypti* receiving infectious WBMs (*p* = 0.0005), indicating viral replication in infected mosquitoes resulted in the dissemination into various tissues from the infected midgut. Although dissemination of ZIKV among mosquitoes receiving infectious BSAMs was also demonstrated by isolation of infectious viruses in secondary tissues, there was no distinguishable difference in the dissemination rates observed at 7 (66.7%, 2/3) and 14 (85.7%, 6/7) d.p.i. (*p* = 1.00). Our observation suggests cellular components in the blood of vertebrate hosts have no demonstrable impact on the dissemination of ZIKV from the midgut of an infected *Ae*. *aegypti*.

**Table 3:** Dissemination rate of ZIKV in *Ae*. *aegypti* receiving infectious blood meals and protein meals at 7 and 14 d.p.i.

Group	7 d.p.i.	14 d.p.i.
WBMs	50.0% (7/14)	100.0% (21/21)
BSAMs	66.7% (2/3)	85.7% (6/7)

**Fig 1:**
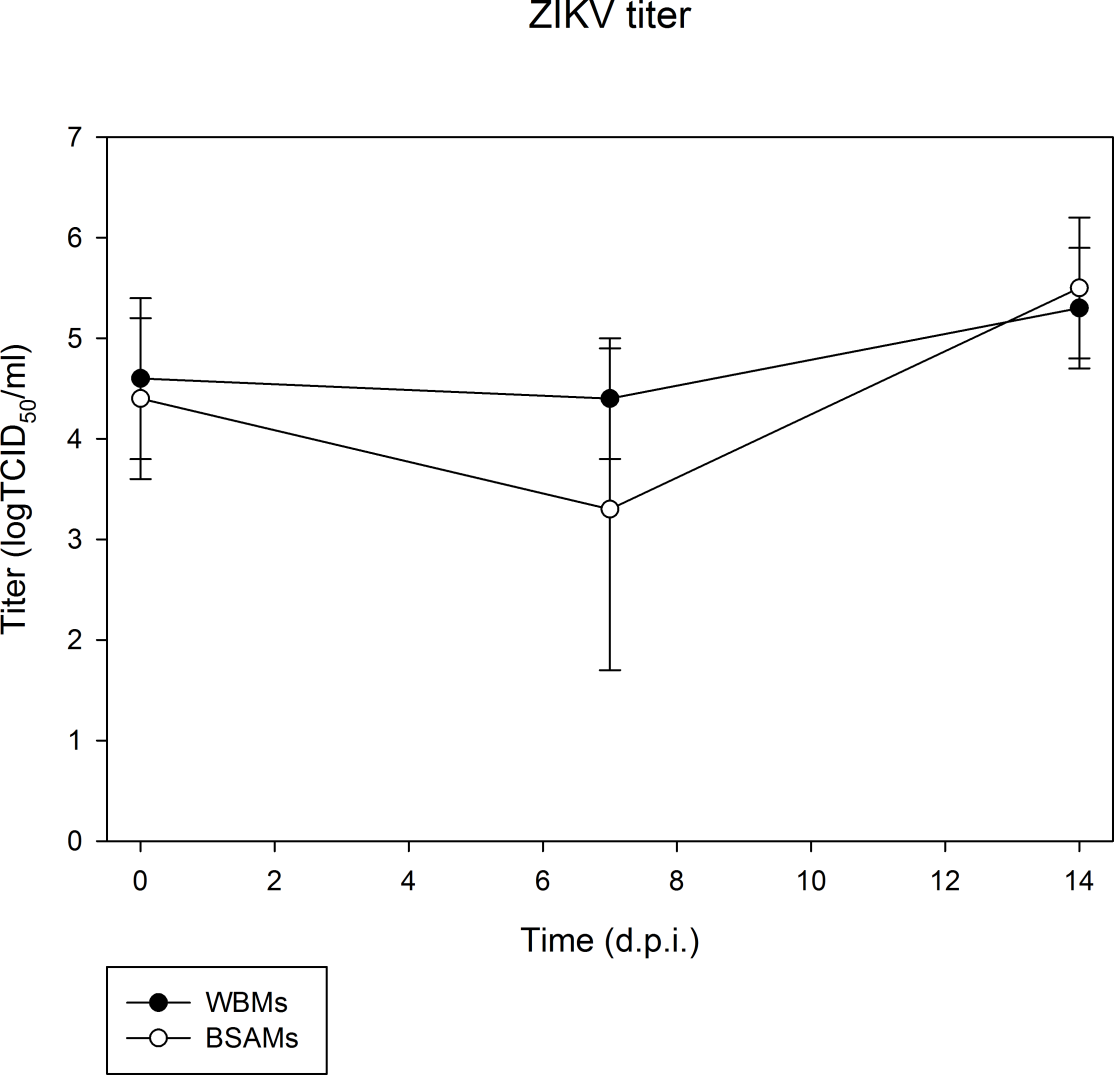
Titers of ZIKV in *Ae*. *aegypti* orally challenged with infectious WBMs and BSAMs. Average titers of infected whole mosquitoes at 0, 7 and 14 d.p.i. from *Ae*. *aegypti* receiving infectious WBMs and BSAMs are shown in solid circle and open circle, respectively.

Titration of infected whole mosquitoes showed comparable titers of ZIKV at both 7 and 14 d.p.i. as shown in Fig 1. The engorgement of ZIKV through infectious WBMs and BSAMs resulted in similar average titers of whole mosquitoes at 4.6±0.8 logTCID_50_/ml and 4.4±0.8 logTCID_50_/ml immediately after feeding at 0 d.p.i., respectively. Although the average titers of infected whole mosquitoes from both groups at 7 d.p.i. showed an insignificant decline compared to the average titers of engorged mosquitoes at 0 d.p.i., there was no demonstrable difference in the average titers of whole mosquitoes in the groups of mosquitoes receiving WBMs (4.4±0.6 logTCID_50_/ml) and BSAMs (3.3±1.6 logTCID_50_/ml) (*p* = 0.06). Similarly, comparable titers of whole mosquitoes were observed between two groups of *Ae*. *aegypti* (WBMs: 5.3±0.6 logTCID_50_/ml; BSAMs: 5.5±0.7 logTCID_50_/ml) at 14 d.p.i. (*p* = 0.60).

The indistinguishable incidence of the disseminated form of infection suggests engorgement of ZIKV through infectious BSAMs only limit the establishment of infection. Viral dissemination driven mostly by viral replication and the production of progeny virions was not affected. This observation was consistent with the lack of significant difference observed in the titers of infected mosquitoes.

## Discussion

Our study demonstrates the differential outcomes in the *per os* challenge of ZIKV between infectious blood meals and infectious protein meals, which have been shown to support the completion of gonotrophic cycles in mosquitoes. Whilst the BSA-based protein meals lead to the similar level of egg production with blood meals in both *Ae*. *aegypti* and *Ae*. *albopictus* [17,18], the oral ingestion of ZIKV in infectious protein meals by *Ae*. *aegypti* leads to significantly lower infection rates than the oral ingestion of ZIKV in infectious whole blood meals. The impact of protein meals in the infection process of ZIKV appeared to be transient and limited to the early phase of infection in our study. There was no demonstrable difference in viral dissemination into secondary tissues and replication kinetics between mosquitoes orally challenged with infectious blood meals and infectious protein meals.

Because albumin and salts are the only ingredients in the formulation of the protein meals used in this study, our results suggest the presence of cellular components in viremic blood may facilitate the establishment of arbovirus infection in *Ae*. *aegypti*. Our reasoning is further supported by a previously published study comparing the infection outcomes of dengue virus serotype 2 after the engorgement of infectious blood meals and substitutive blood meal, which composed of soluble materials in the blood of vertebrate hosts including BSA, λ-globulin, and cholesterol. Although the difference in the infection rates was not reported, the viral load of dengue virus serotype 2 in the midgut of mosquitoes infected through the engorgement of infectious substitutive blood meal was significantly lower than those infected through the engorgement of viremic blood meals at 7 d.p.i. [24]. Results from two independent studies have consistently showed the importance of cellular materials for the infection process of two different flaviviruses. These results are distinct from a recent study demonstrating that there is no demonstrable difference in the infection rates of chikungunya virus through oral exposure of infectious WBMs and BSAMs. The different outcomes of infection through the oral challenges of cell-free infectious protein meals may be a consequence of the fundamental difference in the infection process of mosquitoes between flaviviruses and alphaviruses [25].

It remains unclear which components in the blood of vertebrate hosts are critical for the process of flavivirus infection in mosquitoes. Future studies identifying critical blood components for arbovirus infection may provide important insight on how hematophagy emerged as a mode of transmission for viruses during evolution. More importantly, the characterization of the differential physiological responses induced by blood meals and protein meals may lead to a significant advancement in our knowledge regarding how components of blood can facilitate the infection of arboviruses. The information can ultimately be applicable to the development of tools to disrupt the infection of arboviruses in mosquitoes.
